# A CRISPR/Cas9-based system using dual-sgRNAs for efficient gene deletion in *Mycobacterium abscessus*

**DOI:** 10.3389/fmicb.2025.1608274

**Published:** 2025-07-09

**Authors:** Linai Li, Yuxiang Hu, Dan Wang, Xin Li, Shengjuan Bao, Taibing Deng, Qinglan Wang

**Affiliations:** ^1^Institute of Respiratory Health, Frontiers Science Center for Disease-related Molecular Network, West China Hospital, Sichuan University, Chengdu, China; ^2^School of Clinical Medicine, North Sichuan Medical College, Nanchong, China; ^3^Department of Tuberculosis, Beijing Chest Hospital, Capital Medical University, Beijing, China

**Keywords:** *Mycobacterium abscessus*, CRISPR, dual sgRNAs, genome editing, large fragment deletion

## Abstract

The increasing global prevalence of *Mycobacterium abscessus* infections presents a significant clinical challenge due to the pathogen’s intrinsic resistance to multiple antibiotics and poor treatment outcomes. Despite the necessity of genetic tools for studying its physiology, pathogenesis, and drug resistance, efficient methods for large-fragment deletions remain underdeveloped. Here, we report a CRISPR/Cas9-based dual-sgRNA system employing *Streptococcus thermophilus* CRISPR1-Cas9 (Sth1Cas9), enabling efficient large-fragment knockout in *M. abscessus* with deletion efficiencies exceeding 90% at certain loci and spanning up to 16.7 kb. Furthermore, we systematically optimized the modular arrangement of genetic components in Cas9/dual-sgRNA expression plasmids and refined their construction workflow, achieving a significant reduction in cassette loss rates while enabling single-step plasmid assembly. Notably, deletion efficiency was position-dependent rather than correlated with target size, suggesting an influence of chromatin structure on editing outcomes. As the first CRISPR/Cas9-based platform capable of kilobase-scale deletions in *M. abscessus*, this system advances functional genomics studies and facilitates targeted investigations into virulence and antibiotic resistance mechanisms.

## 1 Introduction

*Mycobacterium abscessus* has emerged as a critical global public health threat, particularly among immunocompromised individuals (van Dorn, 2017). As a rapidly growing nontuberculous mycobacterium (NTM), it causes aggressive pulmonary, cutaneous, and disseminated infections, with clinical treatment failure rates exceeding 50% (Diel et al., 2017). This high failure rate is largely attributed to its multidrug-resistant (MDR) phenotype, including intrinsic resistance to first-line tuberculosis drugs (e.g., isoniazid, rifampin) and inducible macrolide resistance mediated by the *erm(41)* gene (Nessar et al., 2012). Furthermore, *M. abscessus* employs biofilm formation and morphotype switching [smooth (S) to rough (R)] to evade host immune clearance, leading to chronic relapsing infections. Global and regional data indicate a rising burden of pulmonary NTM infections, with a systematic review reporting annual increases of 2.0 cases per 100,000 person-years for infection and 0.5 for disease, and 64.7% of studies showing increased *M. abscessus* infection (Dahl et al., 2022; Cristancho-Rojas et al., 2024). For example, in Catalonia, Spain, NTM-PD prevalence reached 42.8 per 100,000, with a 22% rise in *M. abscessus* isolation and 24% rise in disease between 2003 and 2014 (RR: 1.24, 95% CI: 1.08–1.42; Santin et al., 2018).

Deciphering the biology, pathogenesis, and drug resistance mechanisms of *M. abscessus* necessitates efficient genetic tools. Traditional gene-editing approaches, such as homologous recombination, exhibit low efficiency (10^−6^ to 10^−4^) and are further hampered by high background antibiotic resistance, making mutant screening arduous (Borgers et al., 2019; Chimukuche and Williams, 2021; Medjahed and Reyrat, 2009). The construction of a single-gene knockout strain typically requires screening hundreds to thousands of colonies (Medjahed and Reyrat, 2009), severely limiting functional genomics research. While some studies attempted to mitigate background noise using fluorescent markers (e.g., tdTomato; Viljoen et al., 2018), these modifications failed to fundamentally improve efficiency. Although transposon-based mutagenesis enables genome-wide screening, it lacks precision (Medjahed and Reyrat, 2009).

Recent advances in CRISPR/Cas9 technology offer a transformative alternative for genetic manipulation in *M. abscessus*. CRISPR/Cas9 employs a single guide RNA (sgRNA) to direct Cas9-mediated DNA double-strand breaks (DSBs), inducing frameshift mutations or small indels via non-homologous end joining (NHEJ; Akter et al., 2024; Yan et al., 2020; Neo et al., 2024; Rock et al., 2017; Meijers et al., 2020). The *Streptococcus thermophilus* CRISPR1-derived Cas9 (Sth1Cas9) has demonstrated high editing efficiency in *M. abscessus*, achieving a 10^2^–10^4^-fold improvement over conventional methods (Akter et al., 2024; Neo et al., 2024). Prior studies implemented a dual-plasmid system to mitigate Cas9 cytotoxicity and incorporated fluorescent markers (e.g., mCherry) to enhance screening accuracy (Neo et al., 2024). However, while frameshift mutations effectively inactivate most genes, they may fail to disrupt genes with multiple translation initiation sites and can yield aberrant truncated proteins with unknown physiological consequences. Complete gene deletion is therefore necessary for certain applications.

Here, we report the development of an Sth1Cas9-dual-sgRNA system for highly efficient large-fragment gene knockout in *M. abscessus*. Our approach achieves deletion efficiencies exceeding 90% for some targets, with maximal deletion lengths of 16.7 kb. Notably, knockout efficiency does not correlate with fragment size, suggesting potential genomic location-dependent effects.

## 2 Materials and methods

### 2.1 Bacterial strains and growth conditions

*Mycobacterium abscessus* ATCC 19977 was used as the parental strain for all genetic manipulations. Cultures were grown in Middlebrook 7H9 broth or on Middlebrook 7H10 agar (BD Biosciences, United States) supplemented with 10% OADC (oleic acid, albumin, dextrose, and catalase), 0.2% glycerol (Sangon Biotech), and 0.05% Tween-80 (Sangon Biotech, broth only) at 37°C. Plasmid-containing strains were maintained with the following antibiotics: kanamycin (MCE, United States, 100 μg/mL) for pCas9-mScarlet-containing strains and zeocin (Invitrogen^™^, 20 μg/mL) for pQL033-X-sg-harboring strains. Anhydrotetracycline (aTc; MCE, United States, 500 ng/mL) was used to induce CRISPR components. *Escherichia coli* DH5α (Tsingke; transformation efficiency >10^9^ CFU/μg) was used for plasmid construction and propagation in LB medium (Sangon Biotech) with kanamycin (100 μg/mL) or zeocin (20 μg/mL) as required.

### 2.2 Plasmid construction

To minimize toxicity, the CRISPR/Cas9 system was implemented using two separate plasmids. The Cas9 expression plasmid was derived from pLJR962 (Addgene #115162) via site-directed mutagenesis to introduce A9D and A599H mutations, restoring nuclease activity and generating pCas9. The mScarlet fluorescent reporter was inserted into the EcoRV (New England Biolabs, United States; 10,000 U/mL) site of pCas9 generate pCas9-mScarlet plasmid, allowing visual selection of transformants.

For targeted gene deletion, we developed a dual-sgRNA system based on pKM461 (Addgene #108320). As shown in Supplementary Figure S1, the anhydrotetracycline-inducible promoter (P_tet_), kanamycin resistance gene, and Sth1 sgRNA scaffold (Sth1SC) were amplified from pLJR962 and assembled into pKM461through SapI (New England Biolabs, United States; 10,000 U/mL) and EcoRv sites, generating the pQL033-SapItwin-sgSC plasmid, which incorporated SapI restriction sites for Golden Gate assembly of target-specific sgRNAs. The pUC19-CS-ZeoR plasmid was constructed by inserting the Sth1SC-ZeoR-Ptet cassette into the pUC19 backbone. Target-specific protospacer sequences were designed to flank the genomic region of interest (e.g., Mab_0673/0674) and were incorporated into primers used for amplifying the Sth1SC-ZeoR-Ptet cassette, which contained SapI recognition sites. The resulting PCR product was then cloned into pQL033-SapItwin-sgSC to generate the final dual-sgRNA expression plasmid. All plasmids were confirmed by Sanger sequencing (Tsingke) before use.

### 2.3 Competent cell preparation

*M. abscessus* cells were grown from 1 mL of frozen stock inoculated into 100 mL of Middlebrook 7H9 broth supplemented with 0.2% glycerol, 0.01% Tween-80, and 10% OADC. Cultures were incubated at 37°C, 180 rpm until mid-log phase (OD₆₀₀ = 0.6–0.8), followed by 0.2 M glycine induction for 3 h. Cultures were then pelleted by centrifugation (room temperature, 4,000 rpm, 10 min), and washed sequentially with 30 mL, 20 mL, and 10 mL of 10% glycerol (in deionized water). Cells were finally resuspended in 1 mL of 10% glycerol and ready for electroporation.

### 2.4 Generation of knockout mutants in *Mycobacterium abscessus*

Genetic manipulation of *M. abscessus* was performed using optimized electroporation protocols. Competent cells were prepared as described above. For initial transformation, 1–2 μg of pCas9-mScarlet (or pCas9-mScarlet-noint) plasmid DNA was electroporated using a Bio-Rad Gene Pulser Xcell with parameters set to 2.5 kV, 25 μF, and 1,000 Ω. Transformants were recovered in 7H9/OADC medium for 4 h before plating on selective 7H11/OADC agar containing 100 μg/mL kanamycin. After 4–6 days incubation at 37°C, pink colonies expressing mScarlet were picked for verification by PCR and sequencing of the Cas9 cassette.

To generate knockout mutants, competent cells of the pCas9-mScarlet (or pCas9-mScarlet-noint) strain were transformed with 1 μg of the appropriate pQL033-X-sg plasmid (expressing dual sgRNAs). Following electroporation and recovery, transformants were selected on 7H11 plates containing both kanamycin (100 μg/mL) and zeocin (20 μg/mL). For gene deletion, positive clones were grown to OD600 ~ 0.8 in 7H9 medium with antibiotics, then split into two cultures—one induced with 500 ng/mL aTc and one uninduced control. After overnight induction, serial dilutions were plated on selective media with or without aTc to quantify survival rates. Potential knockout mutants were screened by PCR using primers flanking the target region, with successful deletions identified by the appearance of a smaller amplicon compared to wild-type. The deletion boundaries were confirmed by Sanger sequencing of PCR products. The pQL033-X-sg plasmid was cured by streaking mutant cells onto 7H11/OADC agar supplemented with 5% sucrose in the absence of antibiotics. To remove the pCas9-mScarlet (or pCas9-mScarlet-noint) plasmid from mutant strains, cells were electroporated with plasmid pQL027 (*ZeoR*, *sacB*), which expresses the L5 integrase and excisionase (L5gp36). Transformants were selected on 7H11/OADC agar containing 20 μg/mL zeocin. White colonies indicated loss of the pCas9-mScarlet (or pCas9-mScarlet-noint) plasmid. The pQL027 plasmid was subsequently cured by streaking cells onto 7H11/OADC agar supplemented with 5% sucrose in the absence of antibiotics.

## 3 Results

### 3.1 Design of a dual-sgRNA CRISPR/Cas9 system for gene deletion in *Mycobacterium abscessus*

**Figure 1:**
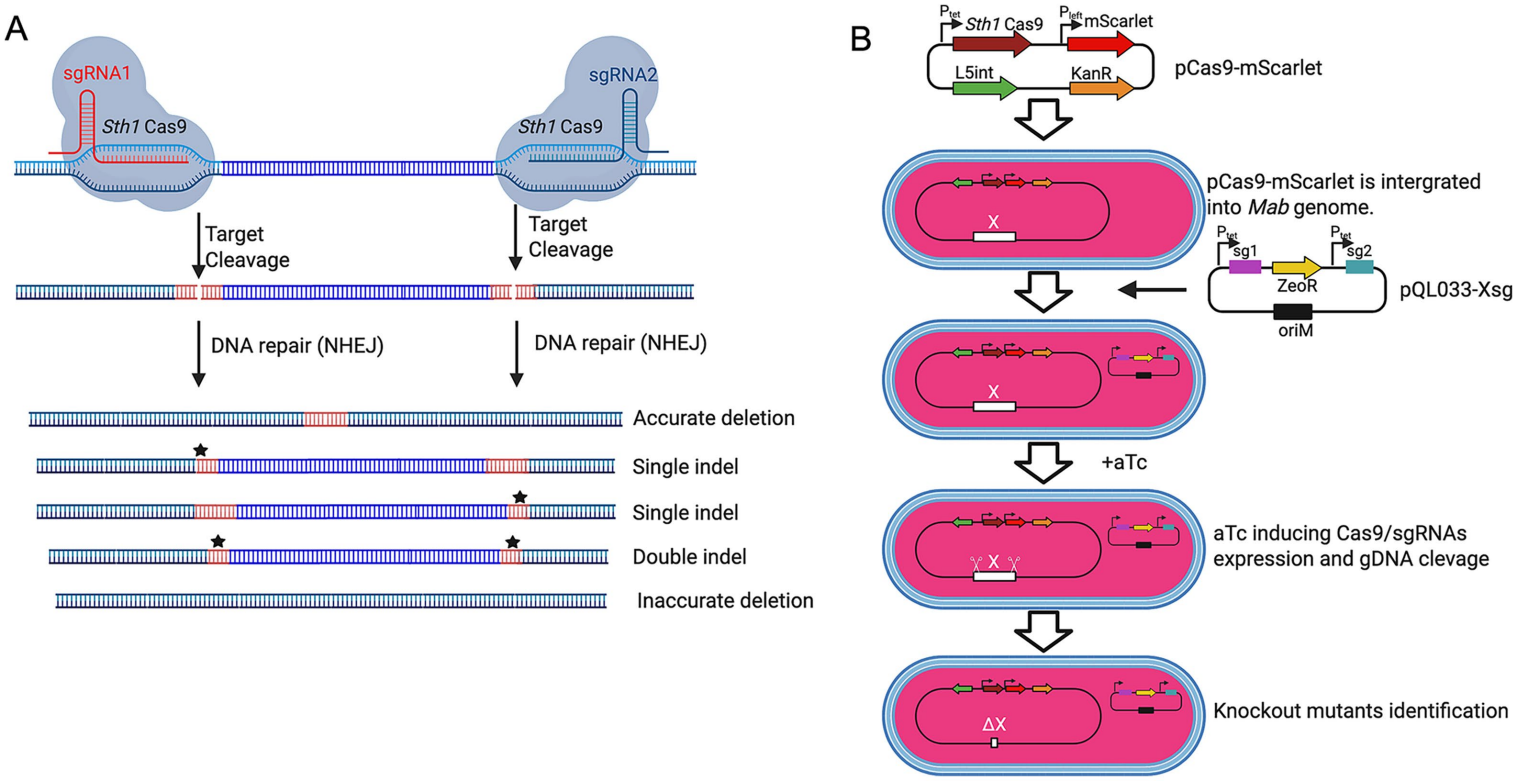
CRISPR-Cas9-mediated gene deletions using dual sgRNAs in *M. abscessus*. **(A)** Schematic of CRISPR-Cas9-induced gene deletions using dual sgRNAs. *M. abscessus* cells harboring pCas9-mScarlet and pQL033-Xsg plasmids express Sth1Cas9 and dual sgRNAs upon aTc induction. The sgRNAs guide Sth1Cas9 to the target genomic loci, inducing double-stranded DNA breaks that can result in precise deletions, single indels, double indels, or inaccurate deletions depending on the repair mechanism. **(B)** Dual-plasmid CRISPR/Cas9 workflow. *M. abscessus* is first transformed with the integrative plasmid pCas9-mScarlet (KanR) encoding inducible Cas9. This Cas9-expressing strain is then transformed with pQL033-Xsg, a plasmid carrying the dual-sgRNA cassette and ZeoR selection marker. CRISPR system activation results in gene deletion and loss of function. All plasmids contain the *E. coli* plasmid replication origin (oriE) to enable plasmid propagation in *E. coli*, but it is omitted from the schematic diagram for clarity.

To establish a CRISPR-based gene knockout system in *M. abscessus*, we engineered a dual-plasmid system comprising a Cas9 expression vector (pCas9-mScarlet) and a dual-sgRNA expressing plasmid (pKMZeoR-sg). The pCas9-mScarlet construct integrates into the L5 attB locus of the *M. abscessus* genome and carries an anhydrotetracycline (aTc)-inducible Sth1Cas9 gene, a kanamycin resistance marker, and an mScarletfluorescent reporter for visual selection of transformants (Figures 1A,B). Following electroporation into wild-type *M. abscessus*, approximately 30% of kanamycin-resistant colonies lacked fluorescence, indicating a high false-positive rate on kanamycin plates.

The sgRNA expression vector (pKMZeoR-sg) is a replicative plasmid conferring zeocin resistance, with sgRNA transcription under tetracycline repressor control. To validate whether dual-sgRNA design could mediate gene knockout in *M. abscessus*, we targeted the *Mab_0673-Mab_0674* (*phoP/phoR*) gene cluster which is of particular interest due to its potential role in virulence regulation, designing two sgRNAs flanking the locus (Supplementary Figures S2A,B). Following electroporation into *M. abscessus* harboring pCas9-mScarlet and aTc induction, no knockout mutants were detected among 69 colonies screened (Supplementary Figure S2C). Sequencing of PCR amplicons from five clones confirmed incomplete cleavage, suggesting inefficient editing. We hypothesized that tandem arrangement of the two sgRNA cassettes might promote intermolecular or intramolecular recombination, leading to sgRNA cassette loss. Indeed, PCR and sequencing of the sgRNA region in three randomly selected clones confirmed extensive deletions in the sgRNA cassettes (Supplementary Figure S2D).

To overcome this limitation, we redesigned the sgRNA expression vector (pQL033-*Mab_0673/0674*sg), by placing the two sgRNA cassettes either side of the ZeoR resistance gene (Supplementary Figures S1, S2E). Electroporation of this modified construct into *M. abscessus* carrying pCas9-mScarlet resulted in significantly improved editing efficiency. PCR analysis 72 colonies showed that 52 colonies had successful *Mab_0673/0674* deletion, 8 lacked deletions, 1 exhibited partial deletion, and 11 produced no PCR amplicon—suggesting larger-than-expected genomic deletions (Supplementary Figure S2F). These findings establish dual-sgRNA CRISPR/Cas9 as an effective strategy for precise gene deletion in *M. abscessus*.

### 3.2 Validation of dual-sgRNA system efficiency in *Mycobacterium abscessus*

**Figure 2:**
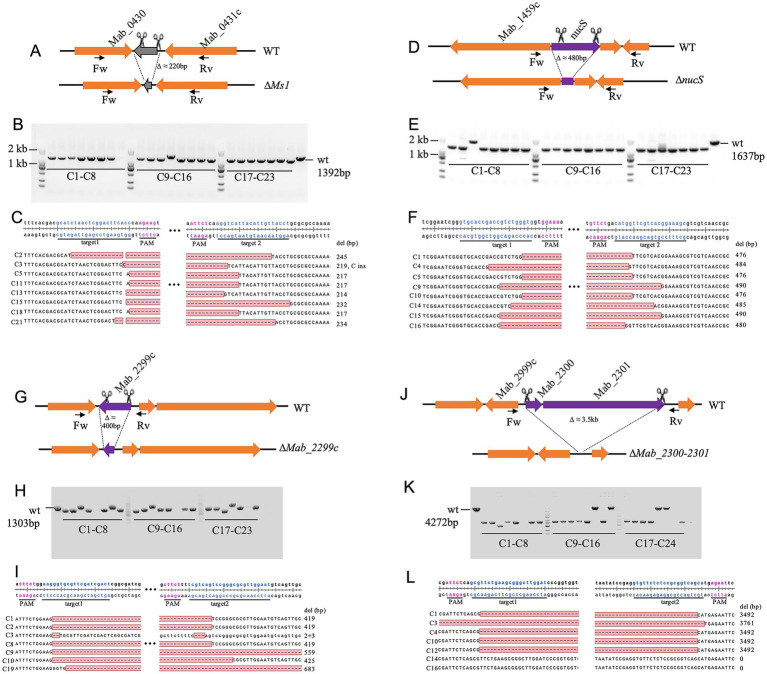
CRISPR-Cas9-mediated deletion of the *Ms1 ncRN*A, *nucS*, *Mab_2999c*, and *Mab_2300/2301* in *M. abscessus*. **(A,D,G,J)** Schematic representation of the deletions of *Ms1 ncRNA*, *nucS*, *Mab_2999c*, and *Mab_2300/2301*, respectively. **(B,E,H,K)** PCR verification of gene knockouts, assessed by agarose gel electrophoresis. For each knockout strain, 23 red colonies were randomly selected for colony PCR validation. Primer pairs used for verification are listed in Supplementary Table S1. **(C,F,I,L)** Sanger sequencing confirmation of deletions in the target genomic regions, with S colonies analyzed per gene.

To systematically assess the gene deletion efficiency of our dual-sgRNA CRISPR–Cas9 system in *M. abscessus*, we targeted four additional loci (*Ms1 ncRNA*, *nucS*, *Mab_2999c*, and *Mab_2300/2301*) distributed across different genomic regions and varying in size (220 bp to 3.5 kb; Figures 2A,D,G,J). Deletion efficiency was consistently high but varied based on genomic context rather than fragment size.

For *Ms1ncRNA* and *nucS* (~200 bp and ~480 bp deletions, respectively), correct knockout rates reached 96% (22/23 clones each) with sequencing confirming precise edits (Figures 2B–F). In contrast, the ~400-bp *Mab_2999c* deletion showed a lower success rate (65%, 15/23 clones), with some exhibiting additional deletions (+140 bp or +264 bp) or loss of PCR amplification (Figures 2G–I). Similarly, the ~3.5 kb deletion of *Mab_2300/2301* was successful in 71% (17/24) of clones, though some exhibited larger deletions (+269 bp) or CRISPR escape events (Figures 2J–L). These results confirm the robustness of the dual-sgRNA system while highlighting potential context-dependent variability in editing efficiency.

### 3.3 Instability of the Sth1Cas9 expression plasmid

**Figure 3:**
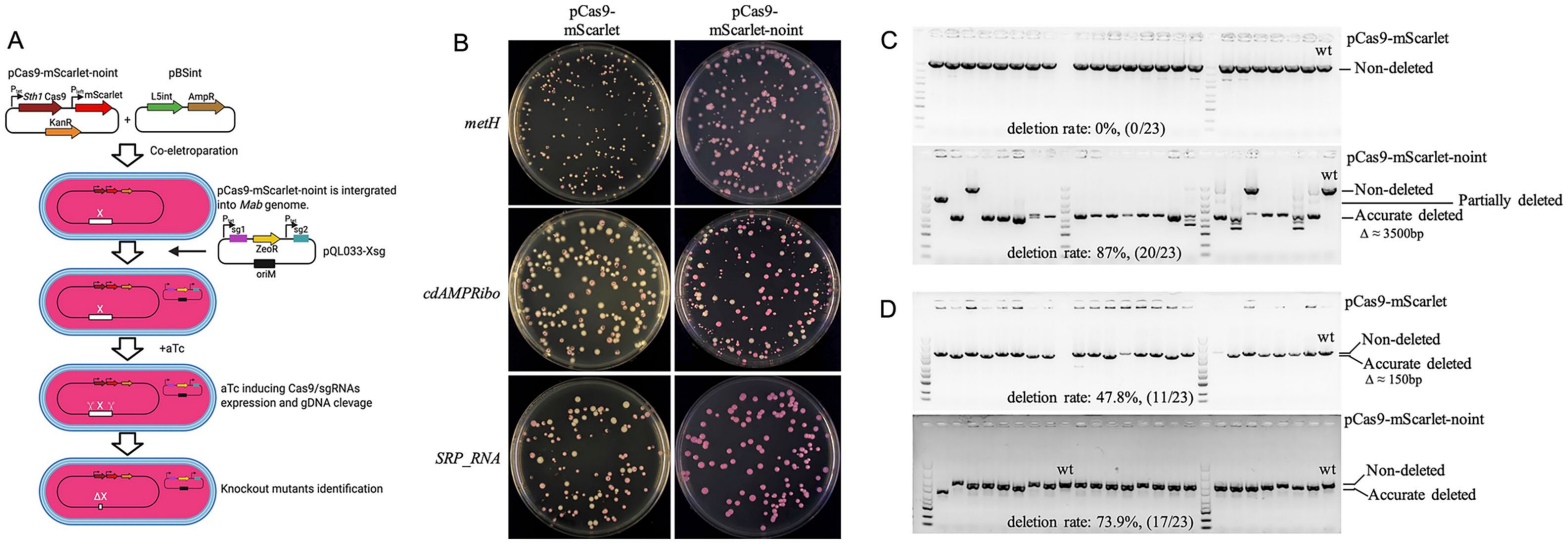
Instability of the pCas9-mScarlet plasmid in *M. abscessus*. **(A)** Schematic diagram of the procedure for constructing *M. abscessus* gene knockout strains using plasmid pCas9-mScarlet-noint. Plasmids pCas9-mScarlet-noint and pBSint were co-electroporated into competent *M. abscessus* cells. Plasmid pBSint transiently expresses the L5 int. integrase, facilitating the integration of pCas9-mScarlet-noint into the bacterial genome. Since plasmid pBSint lacks a mycobacterial origin of replication (oriM), it cannot replicate in *M. abscessus*. Consequently, pBSint is naturally lost during subsequent bacterial cell divisions. The resulting transformants therefore do not contain the L5 int. gene. **(B)** Loss of pCas9-mScarlet or pCas9-mScarlet-noint plasmids during knockout of *metH*, *cdAMPRibo*, and *SRP_RNA* genes. **(C,D)** Deletion efficiency of *metH* **(C)** and *cdAMPRibo* **(D)** validated by colony PCR and agarose gel electrophoresis. For each gene, results are shown for knockouts using the pCas9-mScarlet plasmid (top) and the pCas9-mScarlet-noint plasmid (bottom).

During attempts to delete the methionine synthase gene *metH*, the cyclic di-AMP riboswitch (*cdAMPRibo*, the promoter region of MAB_0869c), and SRP_RNA coding sequence in *M. abscessus*, we observed high rates of pCas9-mScarlet plasmid loss following aTc induction, with white colonies appearing on selective plates (57.1, 67.8, and 20.7%, respectively) (Figure 3A). PCR verification of 23 red colonies revealed no correct knockouts for *metH* and *SRP_RNA*, while only 11/23 clones showed successful *cdAMPRibo* deletion (Figures 3B–D).

Although L5 integration vectors are generally stable in *M. smegmatis* (Saviola, 2009), rare excision events can occur in the presence of L5 integrase. To prevent plasmid loss, we constructed pCas9-mScarlet-noint by removing the L5 integrase gene. Co-electroporation with pBSint (expressing transient L5 integrase) enabled integration of pCas9-mScarlet-noint without persistent integrase expression. This strategy significantly reduced plasmid loss, eliminating white colonies in *metH* and *SRP_RNA* knockouts and reducing loss to 15.5% for *cdAMPRibo* (Figure 3A). PCR verification of randomly selected red surviving clones showed markedly improved knockout efficiency for *metH* and *cdAMPRibo*, reaching 87% (20/23) and 73.9% (17/23) respectively (Figures 3B,C), demonstrating the importance of stabilizing Cas9 expression in some *M. abscessus* genes editing.

### 3.4 Knockout of ultra-long genomic fragments in *Mycobacterium abscessus*

**Figure 4:**
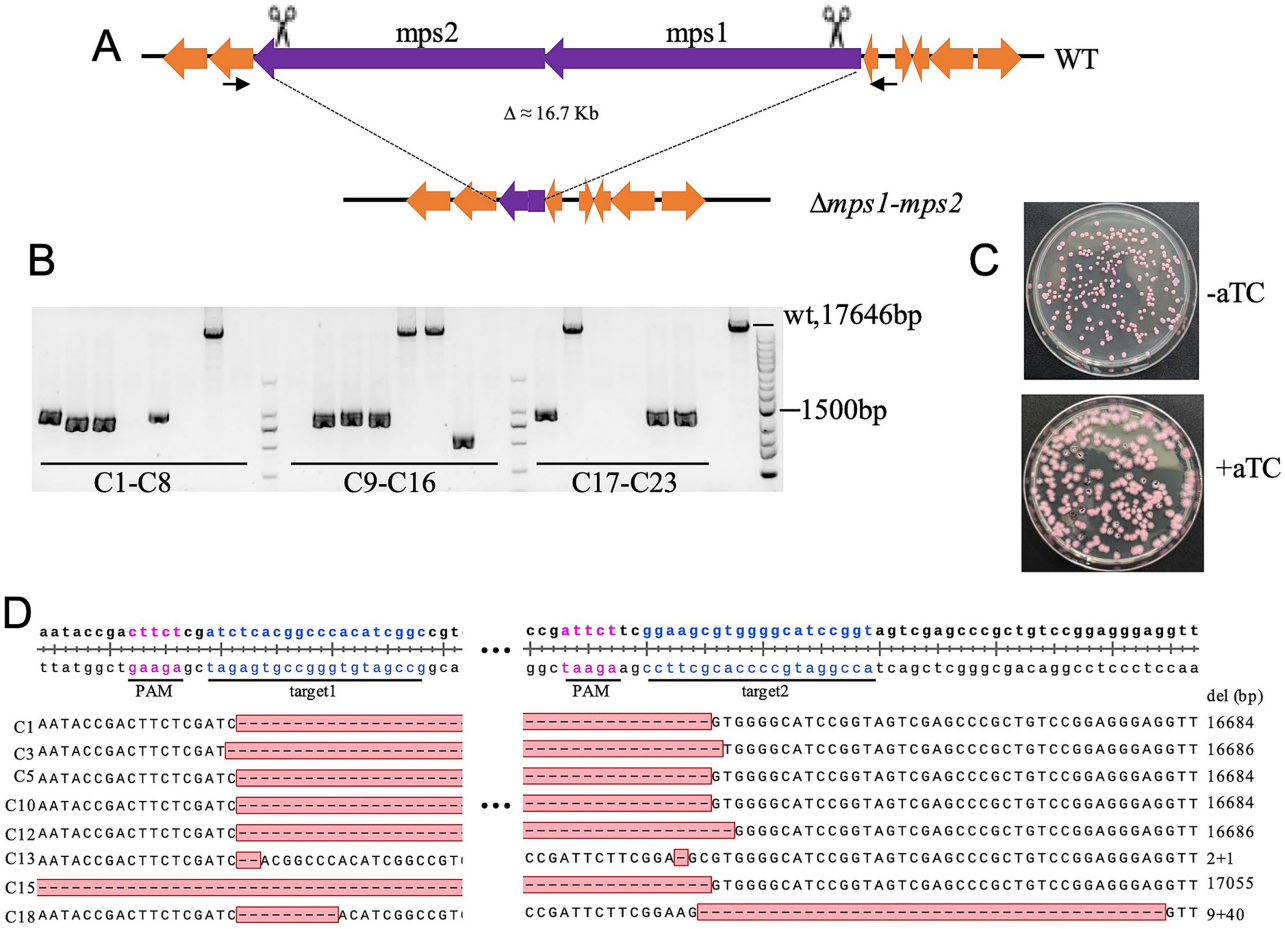
CRISPR-Cas9-mediated deletion of the 16.7-kb *mps1-mps2* fragment in *M. abscessus*. **(A)** Schematic representation of the *mps1-mps2* gene cluster deletion. **(B)** Deletion efficiency validated by colony PCR and agarose gel electrophoresis; 23 red colonies were randomly selected for analysis. **(C)** Colony morphology of *M. abscessus* strains carrying pCas9-mScarlet-noint and pQL033-*mps1-mps2*sg plasmids on plates with or without aTc induction. **(D)** Sanger sequencing confirmation of deletions in the *mps1-mps2* region from eight colonies.

To evaluate the system’s capability for large-scale deletions, we targeted the *mps1-mps2* glycopeptidolipid biosynthetic cluster (18.1 kb), a non-essential region for *in vitro* growth (Figure 4A). Following aTc induction, all surviving clones exhibited rough colony morphology, indicative of *mps1/mps2* inactivation (Figure 4C). PCR screening of 23 rough colonies confirmed successful deletions in 43.5% (10/23), with sequencing verifying an average excision of 16.7 kb (Figures 4B,D). One clone exhibited an additional 370 bp deletion, while eight yielded no PCR products, suggesting larger deletions affecting primer binding sites. Notably, four clones retained wild-type PCR band sizes, and sequencing revealed small deletions near sgRNA target sites (Figure 4D). These findings establish the efficacy of dual-sgRNA CRISPR/Cas9 for ultra-large fragment deletions in *M. abscessus*.

### 3.5 Mab_1080/Mab_1081 encode Msp-like porins whose loss slows, but does not abolish, *Mycobacterium abscessus* growth

Msp-like porins serve as the principal hydrophilic diffusion channels in the outer membrane of rapidly growing mycobacteria (Niederweis, 2003). Partial or complete deletions of the corresponding genes are recurrent in clinical *M. abscessus* isolates and have been linked to increased virulence (Everall et al., 2017; de Moura et al., 2021). de Moura et al. (2021) individually disrupted the two msp homologues, *mmpA* (Mab_1080) and *mmpB* (Mab_1081), in strain CIP108297. Both ∆*mmpA* and ∆*mmpB* mutants grew more slowly *in vitro* than wild type, consistent with a role in nutrient uptake, and each mutant exhibited heightened pathogenicity in SCID mice. Repeated attempts to generate a double knockout with Che9c RecET recombineering failed, leading those authors to propose that at least one functional *mmp* gene is required for *in-vitro* viability.

**Figure 5:**
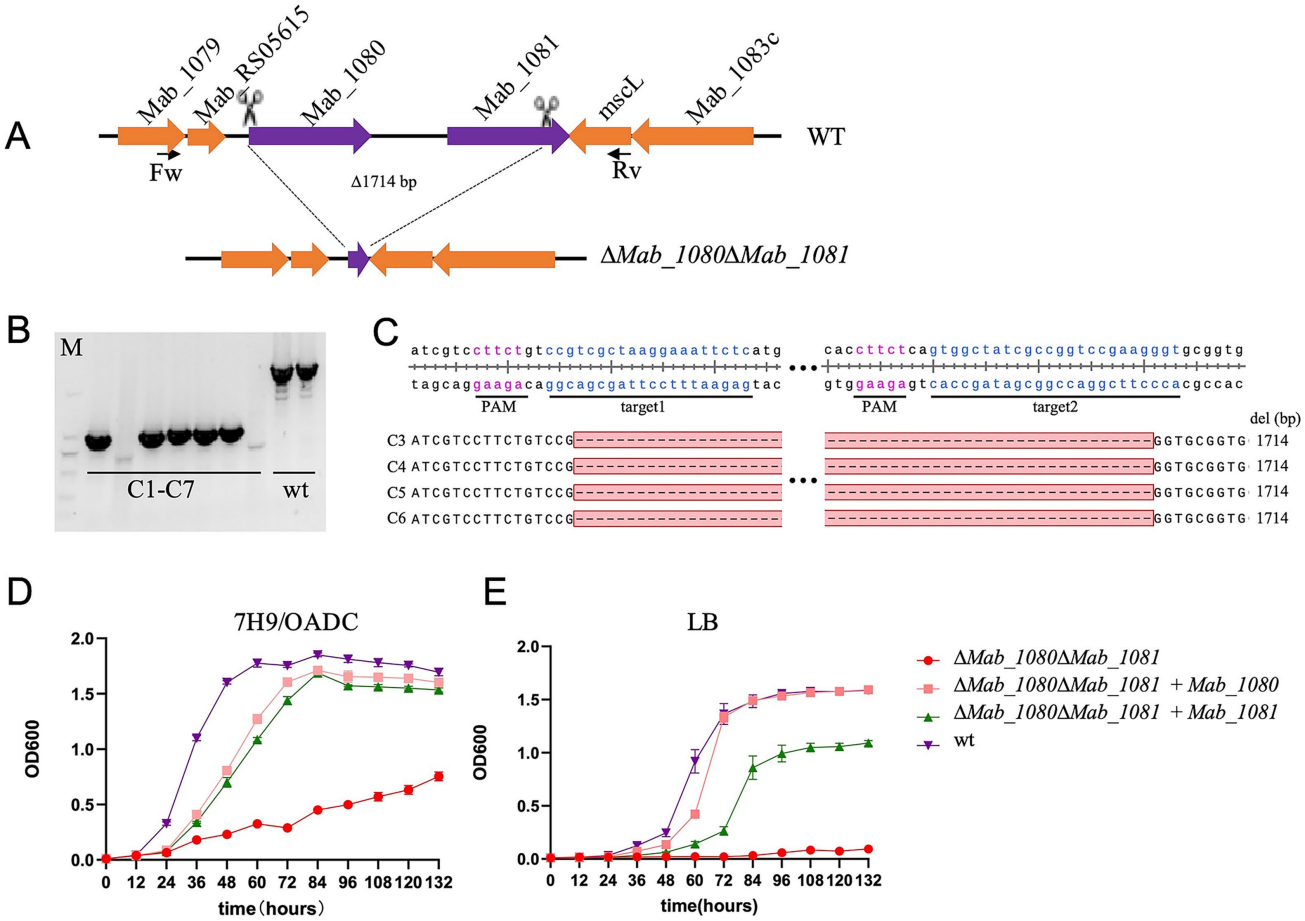
Simultaneous knockout of the outer membrane channel protein genes *Mab_1080/Mab_1081* significantly slowed the growth of *M. abscessus in vitro*. **(A)** Schematic diagram of the construction of the *M. abscessus Mab_1080/Mab_1081* dual-gene knockout strain. **(B)** Verification of the *M. abscessus Mab_1080/Mab_1081* dual-gene knockout strain by PCR and agarose gel electrophoresis. **(C)** Verification of the *M. abscessus Mab_1080/Mab_1081* dual-gene knockout strain (clones 3–6) by clone PCR and Sanger sequencing. **(D,E)** Growth curves of the *M. abscessus* wild-type strain, *Mab_1080/Mab_1081* dual-gene knockout strain, and complemented strains in 7H9/OADC broth **(D)** and LB broth **(E)**. Data are representative of three independent experiments.

Taking advantage of the high efficiency of our dual-sgRNA CRISPR–Cas9 system, we targeted the two adjacent msp-like outer membrane channel genes, *Mab_1080* and *Mab_1081*, for simultaneous deletion in *M. abscessus* strain ATCC 19977. Unexpectedly, we were able to isolate double-knockout (Δ*Mab_1080*Δ*Mab_1081*) mutants on 7H11/OADC agar supplemented with 500 ng/μl aTc (Figures 5A–C). The double-knockout strain exhibited markedly slower growth than the wild type on solid medium, forming visibly smaller colonies over the same incubation period. In 7H9/OADC broth, Δ*Mab_1080*Δ*Mab_1081* mutants displayed a pronounced growth delay relative to wild-type cells. Complementation with either *Mab_1080* or *Mab_1081*, expressed from the P_hsp60_ promoter via the integrative plasmid pMV306, fully restored growth to wild-type levels (Figure 5D). In LB broth, wild-type cells proliferated robustly, whereas the double mutant showed almost no growth; complementation with *Mab_1080* fully rescued proliferation, while *Mab_1081* afforded only partial rescue (Figure 5E).

The ability of the Δ*Mab_1080*Δ*Mab_1081* strain to grow in 7H9/OADC medium, albeit at a reduced rate, indicates that *Mab_1080* and *Mab_1081* are not essential under these conditions. This finding also points to the likely presence of additional outer membrane channel proteins in *M. abscessus*, at least in the ATCC 19977 strain. More broadly, the successful generation of this double knockout underscores the high efficiency of our dual-sgRNA CRISPR system in disrupting genes that strongly impair *in vitro* growth—mutants that are typically difficult to isolate using conventional recombineering approaches.

## 4 Discussion

Gene editing in *M. abscessus* has long been hampered by poor recombination efficiency, largely due to the organism’s low electroporation competency and high frequency of spontaneous drug-resistance mutations. Recently, Akter et al. (2024) and Neo et al. (2024) demonstrated that a CRISPR system employing Sth1Cas9 and a single sgRNA enables efficient site-specific DNA cleavage in the *M. abscessus* genome, which is subsequently repaired by error-prone non-homologous end joining (NHEJ), generating small insertions or deletions (indels) that can inactivate target genes via frameshift mutations. Neo et al. (2024) further attempted to use dual sgRNAs to delete the 324-bp *aar* gene, but they did not succeed in achieving a precise deletion between the two target sites. Instead, they obtained mutant strains with short deletions often extending beyond the intended region.

In contrast, our study introduces key innovations that address the technical limitations observed in prior efforts and establish a reliable platform for efficient and precise genome editing in *M. abscessus*, including large-fragment deletions. By systematically evaluating the dual-sgRNA system across multiple genomic loci and a broad range of deletion sizes—from 220 bp to over16 kb—we demonstrate that precise deletions can be consistently achieved at high efficiency, exceeding 40% even for fragments larger than 16 kb. To our knowledge, this represents the first successful implementation of CRISPR-based large-fragment deletion in *M. abscessus*.

We further identified that the plasmid design used in previous studies may have contributed to their low success rate. Specifically, placing two sgRNA expression cassettes in tandem on a single plasmid promotes homologous recombination between identical promoter, scaffold, or terminator sequences, resulting in frequent loss or truncation of one or both sgRNA modules. To mitigate this, we redesigned the plasmid architecture by separating the two sgRNA cassettes with the *zeoR* antibiotic resistance gene, thereby minimizing homologous regions and stabilizing sgRNA expression. This rearrangement preserved the integrity of both sgRNAs in the vast majority of transformants and likely accounts for the significantly improved editing efficiency observed in our system. To streamline the construction of dual-sgRNA plasmids and support scalable applications, we developed two intermediate vectors, pQL033-SapItwin-sgSC and pUC19-CS-ZeoR, which allow one-step assembly of dual-sgRNA constructs (Supplementary Figure S1). This system greatly reduces the time and labor involved in plasmid construction and provides a practical foundation for high-throughput or genome-wide applications in *M. abscessus*.

Interestingly, we observed no linear inverse correlation between deletion efficiency and fragment size. For instance, deletion of the 3.5-kb *Mab_2300/2301* locus achieved 71% efficiency, whereas deletion of the 150-bp *cdAMPRibo* locus resulted in only 47.8% efficiency. As the protospacers had comparable PAM sequence strength and GC content, this variability is likely due to locus-specific differences in double-strand break (DSB) repair efficiency or spatial constraints between break ends. Future studies targeting additional loci and analyzing larger mutant pools will be required to elucidate the underlying mechanisms influencing deletion efficiency.

To mitigate the cytotoxic effects of simultaneous Cas9 and sgRNA expression—a phenomenon reported previously (Neo et al., 2024)—we separated Cas9 and sgRNA expression into distinct plasmids for sequential transformation. Additionally, an mScarlet reporter, constitutively expressed from the P_left_* promoter, was integrated into the Sth1Cas9 genomic expression plasmid, allowing real-time monitoring of plasmid retention. Notably, certain knockouts, including *metH* and *cdAMPRibo*, resulted in approximately 60% of colonies turning white after aTc induction, suggesting frequent loss of the pCas9-mScarlet plasmid. Red colonies, indicative of plasmid retention, exhibited minimal knockout success, implying a strong counterselection effect at these loci. Removal of the L5 integrase from the Cas9 expressing plasmid significantly reduced the proportion of white colonies, confirming that integrase-mediated excision was the primary loss mechanism. However, residual white colonies in cdAMPRibo knockouts, even with integrase-deficient plasmids, suggest the presence of additional plasmid loss or inactivation pathways. These findings underscore the necessity of mitigating Cas9 plasmid loss when employing Sth1Cas9 for *M. abscessus* genetic manipulation, particularly for constructing CRISPR-based mutant libraries. Strategies such as using integrase-deficient integration plasmids are crucial for maintaining plasmid stability, albeit potentially at the cost of reduced electroporation efficiency when supplemental integrase plasmids are required. Importantly, our study found that *M. abscessus* exhibits low spontaneous resistance to zeocin, suggesting the utility of ZeoR-based selection systems for future genome-editing applications.

In conclusion, we have established a robust, dual-sgRNA CRISPR/Cas9 system for efficient large-fragment deletions in *M. abscessus*. This streamlined, helper-factor-free approach provides a powerful genetic tool for studying bacterial gene function, pathogenicity, and antibiotic resistance in this clinically significant and genetically recalcitrant pathogen.

## Data Availability

The original contributions presented in the study are included in the article/Supplementary material, further inquiries can be directed to the corresponding author.

## References

[ref1] Akter S. Kamal E. Schwarz C. Lewin A. (2024). Gene knock-out in *Mycobacterium abscessus* using *Streptococcus thermophilus* CRISPR/Cas. J. Microbiol. Methods 220:106924. doi: 10.1016/j.mimet.2024.106924, PMID: 38548070

[ref2] Borgers K. Vandewalle K. Festjens N. Callewaert N. (2019). A guide to *Mycobacterium* mutagenesis. FEBS J. 286, 3757–3774. doi: 10.1111/febs.15041, PMID: 31419030

[ref3] Chimukuche N. M. Williams M. J. (2021). Genetic manipulation of non-tuberculosis mycobacteria. Front. Microbiol. 12:633510. doi: 10.3389/fmicb.2021.633510, PMID: 33679662 PMC7925387

[ref4] Cristancho-Rojas C. Varley C. D. Lara S. C. Kherabi Y. Henkle E. Winthrop K. L. (2024). Epidemiology of *Mycobacterium abscessus*. Clin. Microbiol. Infect. 30, 712–717. doi: 10.1016/j.cmi.2023.08.035, PMID: 37778416

[ref5] Dahl V. N. Mølhave M. Fløe A. van Ingen J. Schön T. Lillebaek T. . (2022). Global trends of pulmonary infections with nontuberculous mycobacteria: a systematic review. Int. J. Infect. Dis. 125, 120–131. doi: 10.1016/j.ijid.2022.10.013, PMID: 36244600

[ref6] de Moura V. C. N. Verma D. Everall I. Brown K. P. Belardinelli J. M. Shanley C. . (2021). Increased virulence of outer membrane porin mutants of *Mycobacterium abscessus*. Front. Microbiol. 12:706207. doi: 10.3389/fmicb.2021.706207, PMID: 34335541 PMC8317493

[ref7] Diel R. Ringshausen F. Richter E. Welker L. Schmitz J. Nienhaus A. (2017). Microbiological and clinical outcomes of treating non-*Mycobacterium avium* complex nontuberculous mycobacterial pulmonary disease: a systematic review and meta-analysis. Chest 152, 120–142. doi: 10.1016/j.chest.2017.04.166, PMID: 28461147

[ref8] Everall I. Nogueira C. L. Bryant J. M. Sánchez-Busó L. Chimara E. Duarte R. D. S. . (2017). Genomic epidemiology of a national outbreak of post-surgical *Mycobacterium abscessus* wound infections in Brazil. Microb. Genom. 3:e000111. doi: 10.1099/mgen.0.000111, PMID: 28884021 PMC5562415

[ref9] Medjahed H. Reyrat J. M. (2009). Construction of *Mycobacterium abscessus* defined glycopeptidolipid mutants: comparison of genetic tools. Appl. Environ. Microbiol. 75, 1331–1338. doi: 10.1128/aem.01914-08, PMID: 19114521 PMC2648176

[ref10] Meijers A. S. Troost R. Ummels R. Maaskant J. Speer A. Nejentsev S. . (2020). Efficient genome editing in pathogenic mycobacteria using *Streptococcus thermophilus* CRISPR1-Cas9. Tuberculosis 124:101983. doi: 10.1016/j.tube.2020.101983, PMID: 32829077 PMC7612230

[ref11] Neo D. M. Clatworthy A. E. Hung D. T. (2024). A dual-plasmid CRISPR/Cas9-based method for rapid and efficient genetic disruption in *Mycobacterium abscessus*. J. Bacteriol. 206:e0033523. doi: 10.1128/jb.00335-23, PMID: 38319218 PMC10955840

[ref12] Nessar R. Cambau E. Reyrat J. M. Murray A. Gicquel B. (2012). *Mycobacterium abscessus*: a new antibiotic nightmare. J. Antimicrob. Chemother. 67, 810–818. doi: 10.1093/jac/dkr578 22290346

[ref13] Niederweis M. (2003). Mycobacterial porins—new channel proteins in unique outer membranes. Mol. Microbiol. 49, 1167–1177. doi: 10.1046/j.1365-2958.2003.03662.x 12940978

[ref14] Rock J. M. Hopkins F. F. Chavez A. Diallo M. Chase M. R. Gerrick E. R. . (2017). Programmable transcriptional repression in mycobacteria using an orthogonal CRISPR interference platform. Nat. Microbiol. 2:16274. doi: 10.1038/nmicrobiol.2016.274, PMID: 28165460 PMC5302332

[ref15] Santin M. Barrabeig I. Malchair P. Gonzalez-Luquero L. Benitez M. A. Sabria J. . (2018). Pulmonary infections with nontuberculous mycobacteria, Catalonia, Spain, 1994–2014. Emerg. Infect. Dis. 24, 1091–1094. doi: 10.3201/eid2406.172095, PMID: 29774836 PMC6004863

[ref16] Saviola B. (2009). Phage L5 integrating vectors are present within the mycobacterial cell in an equilibrium between integrated and excised states. Cancer Ther. 7, 35–42. 26316877 PMC4548942

[ref17] van Dorn A. (2017). Multidrug-resistant *Mycobacterium abscessus* threatens patients with cystic fibrosis. Lancet Respir. Med. 5:15. doi: 10.1016/s2213-2600(16)30444-1, PMID: 27956215

[ref18] Viljoen A. Gutiérrez A. V. Dupont C. Ghigo E. Kremer L. (2018). A simple and rapid gene disruption strategy in *Mycobacterium abscessus*: on the design and application of glycopeptidolipid mutants. Front. Cell. Infect. Microbiol. 8:69. doi: 10.3389/fcimb.2018.00069, PMID: 29594066 PMC5861769

[ref19] Yan M. Y. Li S. S. Ding X. Y. Guo X. P. Jin Q. Sun Y. C. (2020). A CRISPR-assisted nonhomologous end-joining strategy for efficient genome editing in *Mycobacterium tuberculosis*. mBio 11:e02364-19. doi: 10.1128/mBio.02364-19, PMID: 31992616 PMC6989103

