# Supplementary material for: A CRISPR/Cas9-based system using dual-sgRNAs for efficient gene deletion in Mycobacterium abscessus
Source: Front Microbiol. 2025 Jul 9;16:1608274. doi: 10.3389/fmicb.2025.1608274 (PMC12283748; doi:10.3389/fmicb.2025.1608274)
Supplement: Supplementary file 1 [file Data_Sheet_1.pdf]

Supplementary Material

1 Supplementary Data

Supplementary Material should be uploaded separately on submission. Please include any supplementary data, figures and/or tables.

Supplementary material is not typeset so please ensure that all information is clearly presented, the appropriate caption is included in the file and not in the manuscript, and that the style conforms to the rest of the article.

2 Supplementary Figures and Tables

For more information on Supplementary Material and for details on the different file types accepted, please see here.

2.1 Supplementary Figures

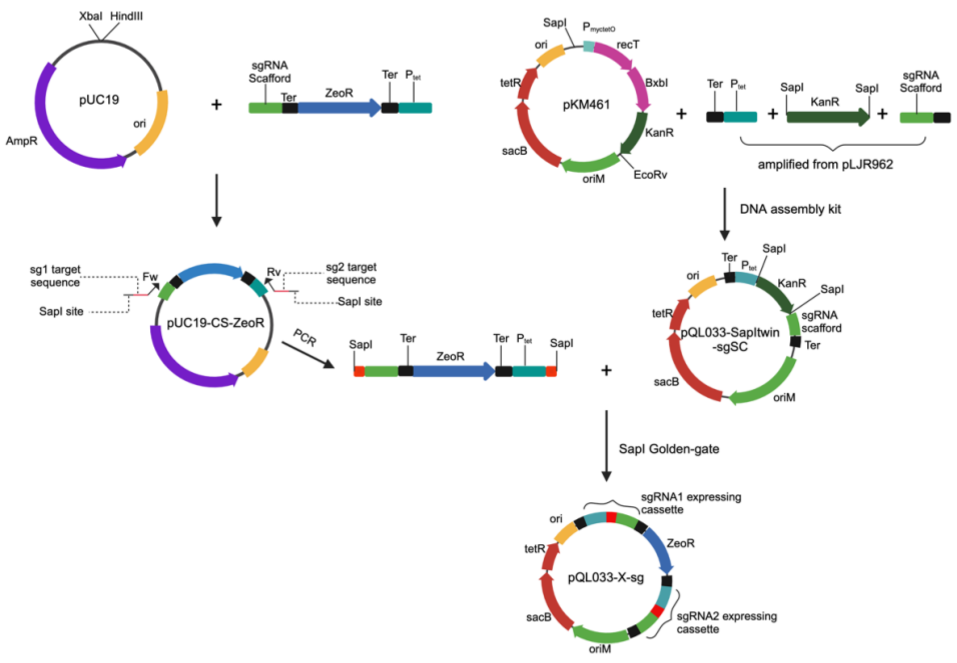

Fig.S1. Schematic of the dual-sgRNA-expressing pQL033-Xsg plasmid construction.

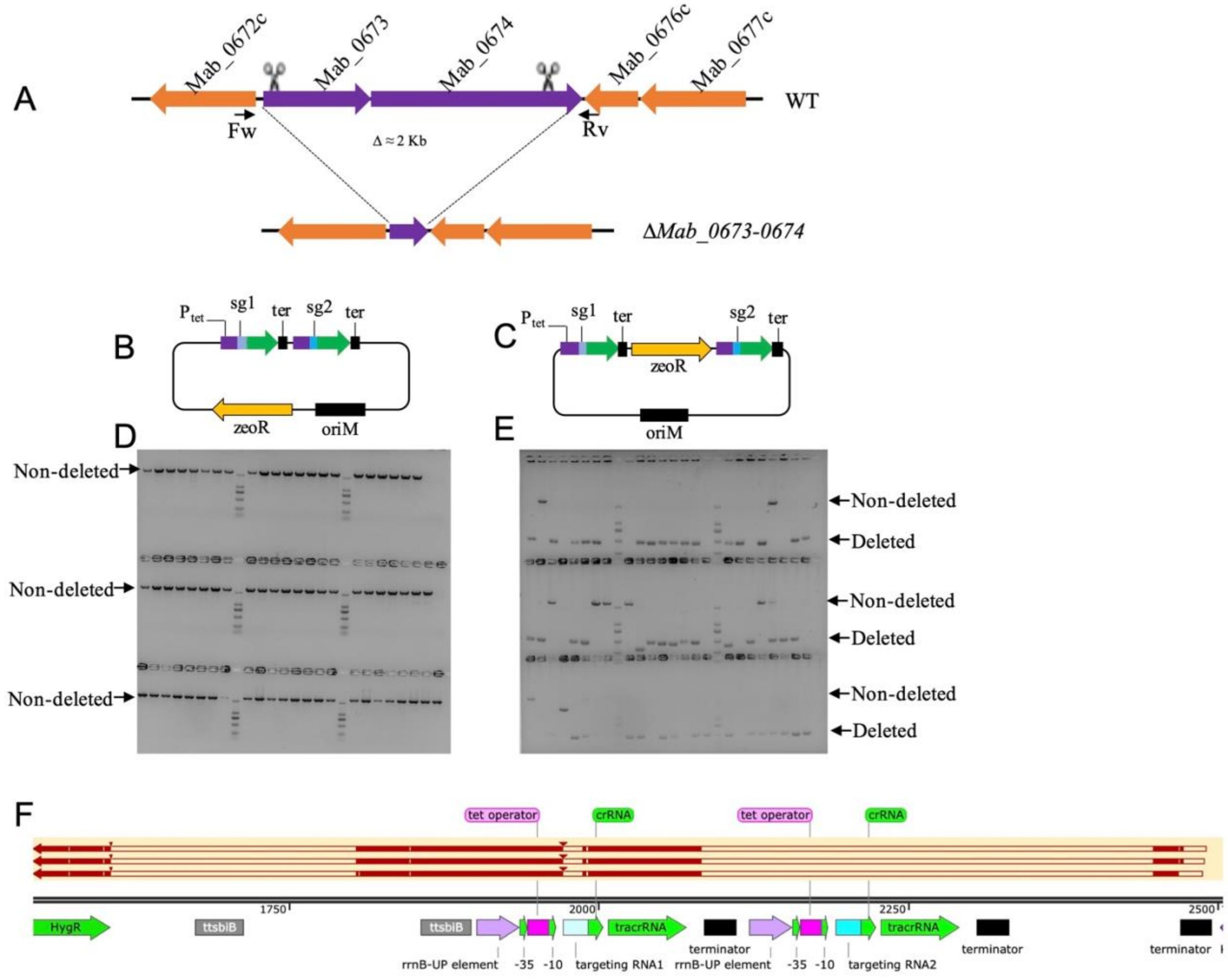

Fig. S2. Comparative analysis of editing efficiencies across dual-sgRNA expression plasmids

(A) Schematic representation of *Mab_0673/0674* gene cluster knockout. (B) Plasmid architecture schematic of pKMZeoR-Mab_0673/074-sg construct. (C) Plasmid architecture of pQL033-Mab_0673/074-sg construct. (D) Colony PCR and agarose gel electrophoresis validation of Mab_0673/074 knockout in M. abscessus strains harboring pCas9-mScarlet and pKMZeoR-Mab_0673/0674-sg plasmids following aTc induction. (E) Colony PCR and agarose gel electrophoresis validation of Mab_0673/0674 knockout in M. abscessus strains carrying pCas9-mScarlet and pQL033-Mab_0673/0674-sg plasmids after aTc induction. (F) Loss of the sgRNA expression cassette in the pKMZeoR-Mab_0673/0674-sg plasmid, assessed by PCR amplification and Sanger sequencing of the sgRNA cassette region in three randomly selected clones from panel D. The central thick double solid line represents the template sequence of the pKMZeoR-

Mab_0673/0674-sg plasmid. The colored boxes below represent the sequences of different genetic elements (identical colors indicate identical genetic element sequences). The three red bars above the double solid line represent the alignment results of Sanger sequencing for the sgRNA cassette region from three clones compared to the original plasmid sequence. Solid red bar segments indicate regions retained in the clone, while hollow red bar segments indicate deleted regions.

2.2 Supplementary Tables

Table S1. Oligonucleotides used in this study. Restriction sites are underlined (SapI), OVERhang regions are italicized, CRISPR-Cas9 sgRNA targeting regions are Bolded.

#	Primer name	Sequence(5' → 3')
1	MabHeesg1_FW_SapIGGA	GGCTACGCTCTTCG*GGA* **AGGTAACAATGTAATGACCT** GTTTTTGTACTCGAAAGAAGCTACAAAGA
2	MabHeesg2_Rv_SapIGTT	GGCTACGCTCTTCG*AAC* **GGTGAAGTCCGAGTTAGATGC** TCCCAGATTATATCTATCACTGATAGGGAT
3	MabnucSsg1_FW_SapIGGA	GGCTACGCTCTTCG*GGA* **GTGCACCGACCGTCTGGGTG** GTTTTGTACTCGAAAGAAGCTACAAAGA
4	MabnucSsg2_Rv_SapIGTT	GGCTACGCTCTTCG*AAC* **CATGGTTCGTCACGGAAAGC** TCCCAGATTATATCTATCACTGATAGGGAT
5	MabNcRNA1sg1_FW_SapIGGA	GGCTACGCTCTTCG*GGA* **ACCTCGCAGGTGCGTCACAGA** GTTTTTGTACTCGAAAGAAGCTACAAAGA
6	MabNcRNA1g2_Rv_SapIGTT	GGCTACGCTCTTCG*AAC* **CGCGGTCCACACCCGAGGGAC** TCCCAGATTATATCTATCACTGATAGGGAT
7	MabNcRNA2sg1_FW_SapIGGA	GGCTACGCTCTTCG*GGA* **ACCCCCACCACAGTTAACGCTGGGG** GTTTTTGTACTCGAAAGAAGCTACAAAGA
8	MabNcRNA2g2_Rv_SapIGTT	GGCTACGCTCTTCG*AAC* **AGATCGCGCCTCCGGATGCC** TCCCAGATTATATCTATCACTGATAGGGAT

9	MabmetHsg1_FW_SapIGGA:	GGCTACGCTCTTCG*GGA***AGTTAAGGACGCCTTCCGCT**GTTTTTGTACTCGAAAGAAGCTACAAAGA
10	MabmetHsg2_Rv_SapIGTT	GGCTACGCTCTTCG*AAC***GTGTGCTCGGGGCAGGCCGGGT**TCCCAGATTATATCTATCACTGATAGGGAT
11	Mab_2299csg1_FW_SapIGGA	GGCTACGCTCTTCG*GGA***AGTCGATCGAACGCACCCTT**GTTTTTGTACTCGAAAGAAGCTACAAAGA
12	Mab_2299csg2_Rv_SapIGTT	GGCTACGCTCTTCG*AAC***ATTCCAACGCGCCCGGACTGACGA**TCCCAGATTATATCTATCACTGATAGGGAT
13	Mab_2300_2301sg1_FW_SapIGGA	GGCTACGCTCTTCG*GGA***ATCCAAGCCCGCTTCAGAACGC**GTTTTTGTACTCGAAAGAAGCTACAAAGA
14	Mab_2300_2301sg2_Rv_SapIGTT	GGCTACGCTCTTCG*AAC***TGCTGACCGCGGAGAGAACAC**TCCCAGATTATATCTATCACTGATAGGGAT
15	Mab_0673sg1_FW_SapIGGA_1:	**GCGGGGGTGGTGCCGTTGGC**GTTTTTGTACTCGAAAGAAGCTACAAAGA
16	Mab_0673sg1_FW_SapIGGA_2	GGGCTACGCTCTTCG*GGA***GCGGGGGTGGTGCCGTTGGC**GTT
17	Mab_0674sg2_Rv_SapIGTT_1	**CGCGCGGATTCCTCGCGCAC**TCCCAGATTATATCTATCACTGATAGGGAT
18	Mab_0674sg2_Rv_SapIGTT_2	GGCTACGCTCTTCG*AAC***CGCGCGGATTCCTCGCGCAC**T
19	MabMps1sg1_FW_SapIGGA_1	**ACCGGATGCCCCACGCTTCC**GTTTTTGTACTCGAAAGAAGCTACAAAGA
20	MabMps1sg1_FW_SapIGGA_2	GGCTACGCTCTTCG*GGA***ACCGGATGCCCCACGCTTCC**GTT
21	MabMps2sg2_Rv_SapIGTT_1	**ATCTCACGGCCCACATCGGC**TCCCAGATTATATCTATCACTGATAGGGAT
22	MabMps2sg2_Rv_SapIGTT_2	GGCTACGCTCTTCG*AAC***ATCTCACGGCCCACATCGGC**T

23	Mab_1080sg1_FW_SapIGGA_1	**GAGAATTTCCTTAGCGACGG**GTTTTTGTACTCGAAAGAAGCTACAAAGA
24	Mab_1080sg1_FW_SapIGGA_2	GGCTACGCTCTTCG*GGA***GAGAATTTCCTTAGCGACGG**
25	Mab_1081sg2_Rv_SapIGTT_1	**GTGGCTATCGCCGGTCCGAAGGGT**TCCCAGATTATATCTATCACTGATAGGGAT
26	Mab_1081sg2_Rv_SapIGTT_2	GGCTACGCTCTTCG*AAC***GTGGCTATCGCCGGTCCGAAGGGT**
27	MABHeeKOcon_FW	ATGGCAGATGAGGACACC
28	MABHeeKOcon_Rv	ATGTGGAACAAGTGAAGGC
29	MABnucSKOcon_FW	GGACTATGACGAAGTTGAC
30	MABnucSKOcon_Rv	GAGGCCATGCTGTCCTTT
31	MABNcRNA1KOcon_FW	ATCGACGAGATGGCTGTG
32	MABNcRNA1KOcon_Rv	AGCATCACGGTCAGGTAT
33	MABmetHKOcon_FW	AGCAAGCGGTTGCAGAAC
34	MABmetHKOcon_Rv	CCATCGCACCAGATGAGA
35	MAB2299cKOcon_FW	TTAGCGGGCATCGGGTTG
36	MAB2299cKOcon_Rv	CGAACCTGGCTCAACTACCG

37	MAB2300_2301KOcon_FW	GCGTTGGAATGTCAGTTGCG
38	MAB2300_2301KOcon_Rv	GTTCACCGCCCTAAGCAC
39	MAB0673_74KOcon_FW	CCAATCCGGTGTCGGCAA
40	MAB0673_74KOcon_Rv	CGCACGATCTTTGGTTCGGA
41	MABmps1_2KOcon_FW	GATTCATCACGTGGTAGGTCTC
42	MABmps1_2KOcon_Rv	GTTCGGATCGAAGGTCAAGTAG
43	MAB1080_81KOcon_FW	CACGCTGGTGGTCAACAT
44	MAB1080_81KOcon_Rv	GCTGGTCACCAAGTTCAGTGA
